# C-peptide and Retinal Microangiopathy in Diabetes

**DOI:** 10.1080/15438600490424569

**Published:** 2004

**Authors:** Subrata Chakrabarti, Zia Ali Khan, Mark Cukiernik, Weixian Zhang, Anders A. F. Sima

**Affiliations:** 1 Department of Pathology University of Western Ontario London Ontario Canada; 2 Department of Pathology and Neurology Wayne State University Detroit Michigan USA; 3 FRCPC University of Western Ontario Department of Pathology Health Sciences Addition, 1151 Richmond Street North London ON N6A 5C1 Canada

## Abstract

Increased extracellular matrix (ECM) protein deposition and capillary basement membrane (BM) thickening are characteristic features of diabetic retinal microangiopathy. Recent observations in the authors' laboratories suggest that high glucose in endothelial cells as well as diabetes causes up-regulation of total fibronectin (FN), as well as extradomain-B (EDB) containing the spliced variant of FN, oncofetal FN, in the retina. This splice variant is normally absent in mature adult tissues and is believed to be involved in angiogenesis. In this study, the authors have investigated the role of C-peptide in the production of ECM proteins and capillary BM thickening in the retina of diabetic rats. They investigated retinas from poorly controlled diabetic BB/Wor rats with or without C-peptide treatment as well as those from age-matched nondiabetic control rats after 8 months of diabetes. In addition, the authors investigated retinas from BBDRZ/Wor rats, a model of type 2 diabetes. Following a treatment period of 8 months, retinal tissues were harvested for gene expression and histological analyses. In the retinas of diabetic BB/Wor rats, a significant increase of oncofetal FN was demonstrated compared to control rats. C-peptide treatment of BB/Wor rats completely prevented such increase. Furthermore, retinas from BBDRZ/Wor rats, did not exhibit any such alteration in oncofetal FN expression. The authors further examined retinal capillary BM thickening using ultrastructural morphometry. C-peptide treatment was ineffective in preventing the diabetes-induced increase in capillary BM thickness. The authors' previous studies of cultured endothelial cells demonstrated that oncofetal FN synthesis is, at least in part, mediated via transforming growth factor-*β* (TGF-*β*) and endothelin-1 (ET-1). Hence, they examined these two transcripts in the retina of these animals. Diabetes caused significant increase in mRNA expression of ET-1 and TGF-*β*, which was not prevented by C-peptide treatment. Hence it appears that C-peptide is effective in preventing diabetes-induced oncofetal FN expression and that these effects are not mediated via ET-1 or TGF-*β*. In conclusion, these data suggest that C-peptide is involved in regulating ECM protein composition. Furthermore, normalization of diabetes-induced oncofetal FN up-regulation may suggest importance of C-peptide in advanced alterations in diabetic retinopathy such as angiogenesis.


*Experimental Diab. Res.*, 5:91–96, 2004
Copyright © Taylor and Francis Inc.
ISSN: 1543-8600 print / 1543-8619 online
DOI: 10.1080/15438600490424569

Taylor & Francis
Taylor & Francis Group

### C-peptide and Retinal Microangiopathy in Diabetes

**Subrata Chakrabarti,[1] Zia Ali Khan,[1] Mark Cukiernik,[1] Weixian Zhang,[2] and Anders A. F. Sima[2]**

[1]*Department of Pathology, University of Western Ontario, London, Ontario, Canada*
[2]*Department of Pathology and Neurology, Wayne State University, Detroit, Michigan, USA*

**Increased extracellular matrix (ECM) protein deposition and capillary basement membrane (BM) thickening are characteristic features of diabetic retinal microangiopathy. Recent observations in the authors' laboratories suggest that high glucose in endothelial cells as well as diabetes causes up-regulation of total fibronectin (FN), as well as extradomain-B (EDB) containing the spliced variant of FN, oncofetal FN, in the retina. This splice variant is normally absent in mature adult tissues and is believed to be involved in angiogenesis. In this study, the authors have investigated the role of C-peptide in the production of ECM proteins and capillary BM thickening in the retina of diabetic rats. They investigated retinas from poorly controlled diabetic BB/Wor rats with or without C-peptide treatment as well as those from age-matched nondiabetic control rats after 8 months of diabetes. In addition, the authors investigated retinas from BBDRZ/Wor rats, a model of type 2 diabetes. Following a treatment period of 8 months, retinal tissues were harvested for gene expression and histological analyses. In the retinas of diabetic BB/Wor rats, a significant increase of oncofetal FN was demonstrated compared to control rats. C-peptide treatment of BB/Wor rats completely prevented such increase. Furthermore, retinas from BBDRZ/Wor rats, did not exhibit any such alteration in oncofetal FN expression. The authors further examined retinal capillary BM thickening using ultrastructural morphometry. C-peptide treatment was ineffective in preventing the diabetes-induced increase in capillary BM thickness. The authors' previous studies of cultured endothelial cells demonstrated that oncofetal FN synthesis is, at least in part, mediated via transforming growth factor-$\beta$ (TGF-$\beta$) and endothelin-1 (ET-1). Hence, they examined these two transcripts in the retina of these animals. Diabetes caused significant increase in mRNA expression of ET-1 and TGF-$\beta$, which was not prevented by C-peptide treatment. Hence it appears that C-peptide is effective in preventing diabetes-induced oncofetal FN expression and that these effects are not mediated via ET-1 or TGF-$\beta$. In conclusion, these data suggest that C-peptide is involved in regulating ECM protein composition. Furthermore, normalization of diabetes-induced oncofetal FN up-regulation may suggest importance of C-peptide in advanced alterations in diabetic retinopathy such as angiogenesis.**

**Keywords** Basement Membrane; C-Peptide; Diabetes; Fibronectin; Oncofetal Fibronectin; Retina

### INTRODUCTION

Long-term complications of diabetes are as debilitating as the disease itself. Patients with diabetes develop secondary complications, such as nephropathy, neuropathy, and retinopathy (Brownlee, 2001). Diabetic retinopathy (DR) progresses through the background, to preproliferative, and finally the proliferative stages (Sheetz and King, 2002). Hyperglycemia is associated with the initiation as well as the progression of DR (The Diabetes Control and Complications Trial Research Group, 1993; UK Prospective Diabetes Study Group, 1998). Several biochemical abnormalities are associated with hyperglycemia, including increases in oxidative stress, nonenzymatic glycation of proteins, activation of the polyol pathway, and

Received 2 October 2003; accepted 29 November 2003.

The authors acknowledge grant supports from the Canadian Diabetes Association, in honor of Margaret Francis (SC), Canadian Institutes of Health Research (SC), and Internal Research Fund of the Lawson Health Research Institute (SC), the Juvenile Diabetes Research Foundation (AAFS), and Thomas Foundation (AAFS).

Address correspondence to Subrata Chakrabarti, PhD, MD, FRCPC, University of Western Ontario, Department of Pathology, Health Sciences Addition, 1151 Richmond Street North, London, ON, Canada N6A 5C1. E-mail: schakrab@uwo.ca up-regulation of growth factors, vasoactive peptides, and cytokines (Cai and Boulton, 2002). Pathologically, the result of such biochemical changes include the loss of retinal capillary pericytes, which leads to the formation of capillary ghosts, the thickening of retinal capillary basement membrane (BM), and neovascularization (Tsilibary, 2003). Capillary BM thickening is one of the hallmarks of diabetic retinal microangiopathy (Tsilibary, 2003). We have previously demonstrated that BM thickening is accompanied by increased alpha-1 (IV) collagen and fibronectin expression (Evans et al., 2000).

Fibronectin (FN) is one extracellular protein that is up-regulated due to diabetes. FN is a dimeric protein produced as a soluble form, which is secreted into the blood (plasma FN), and a nonsoluble isoform, which is cross-linked and deposited within the extracellular matrix (ECM) (cellular FN) (Kornblihtt et al., 1996). The FN dimer is composed of nonidentical polypeptides of 250 to 280 KDa, made up of type I (40 amino acid [aa] residues), type II (60 aa residues), and type III (90 aa residues) repeats. The single rat and human FN gene can produce several isoforms (12 in the rat, 20 in humans) by way of altenative splicing (Schwarzbauer et al., 1983; Kornblihtt et al., 1984).

Within the FN gene, an area of variability (EDB or EIIIB) encodes a single exon that translates to a highly conserved type III repeat (Zardi et al., 1987). This EDB-encoded area is not found in plasma FN, but is found in embryonic tissue and transformed cells. Hence, this FN isoform is known as oncofetal FN (Carnemolla et al., 1989; Zardi et al., 1987). We have recently demonstrated that oncofetal FN is up-regulated both in human and animal diabetes (Khan et al., 2004).

Along with hyperglycemia, type 1 diabetes show a lack of insulin and C-peptide. C-peptide is the spacer protein that is cleaved from proinsulin, allowing the correct conformational folding of insulin for secretion and interaction with the insulin receptor (Wahren et al., 2000). Initially believed to possess no other biological role than insulin synthesis (Kitabchi, 1977), the opinion of C-peptide as a biologically inactive molecule has changed based on recent research. C-peptide treatment leading to increased $Na^+/K^+$-ATPase activity, mitogen-activated protein kinase (MAPK) activity, $Ca^{2+}$ influx, endothelial nitric oxide synthase (eNOS) activity, phosphoinositide 3-kinase (PI3K), and protein kinase C (PKC) activity has been demonstrated in the context of diabetes (Grunberger et al., 2001; Johansson et al., 2002; Li et al., 2001, 2003; Wahren et al., 2000).

Animal experiments with C-peptide–treated streptozotocin (STZ)-induced diabetic rats have resulted in improved glomerular hyperfiltration and decreased protein leakage (Samnegard et al., 2001; Sjoquist et al., 1998). Similar data have been demonstrated in human diabetes (Johansson et al., 1992). STZ-diabetic animals treated with C-peptide showed improvements in diabetes-induced reduced motor nerve conduction velocity and reduced sciatic nerve $Na^+,K^+$-ATPase activity (Cameron et al., 2001; Ido et al., 1997; Sima et al., 2001). In the type I BB/Wor rat, C-peptide replacement prevented small and large fiber functional deficits as well as structural changes of peripheral nerve (Sima et al., 2001; Sima, 2003; Pierson et al., 2003). In the retina, C-peptide treatment has been shown to improve retinal blood flow and reduce vascular permeability (Ido et al., 1997). However, no data are available as to the possible role of C-peptide in ECM protein production and subsequent capillary BM thickening.

In this report, we determined the effects of long-term C-peptide treatment on the expression of the ECM proteins, FN, and oncofetal FN, and capillary BM thickening in the retina of the diabetic BB/Wor rat.

### C-PEPTIDE AND FIBRONECTIN EXPRESSION

Using specific and sensitive real time reverse transcriptase–polymerase chain reaction (RT-PCR) assay (Chen et al., 2003), we have investigated whether C-peptide regulates expression of FN and oncofetal FN. RNA was extracted from frozen retinal tissues and subjected to RT-PCR as previously described (Chen et al., 2003). Retinas of male BB/Wor diabetic rats after 8 months of diabetes exhibited increased expression of total FN as well as relative oncofetal FN (oncofetal FN:total FN) as compared to age and sex-matched nondiabetic controls (Figure 1). Interestingly, C-peptide treatment (75 ng/kg/day; Pierson et al., 2003) of BB/Wor rats completely normalized the diabetes-induced oncofetal FN up-regulation (Figure 1). Further, in support of the potential role of C-peptide in ECM protein alterations, retinas from BBDRZ/Wor rats (model of type II diabetes with normal C-peptide levels) showed no alteration in oncofetal FN expression levels.

### C-PEPTIDE REGULATES ECM PROTEIN EXPRESSION INDEPENDENT OF TGF-$\beta$1 AND ET-1

Our previous studies have demonstrated that glucose-induced oncofetal FN expression in vascular endothelial cells is, in part, mediated via transforming growth factor-$\beta$ (TGF-$\beta$) and endothelin-1 (ET-1) (Khan et al., 2004). In addition, TGF-$\beta$ has been shown to regulate the preferential expression of oncofetal FN in various tissues during remodeling and wound healing (Jarnagin et al., 1994; Nickeleit et al., 1996). We determined whether these potent mitogenic growth factors are transducers of C-peptide signals in regulating oncofetal FN in retinas of C-peptide–treated diabetic animals. Retinal tissues of BB/Wor diabetic animals showed significantly increased mRNA levels

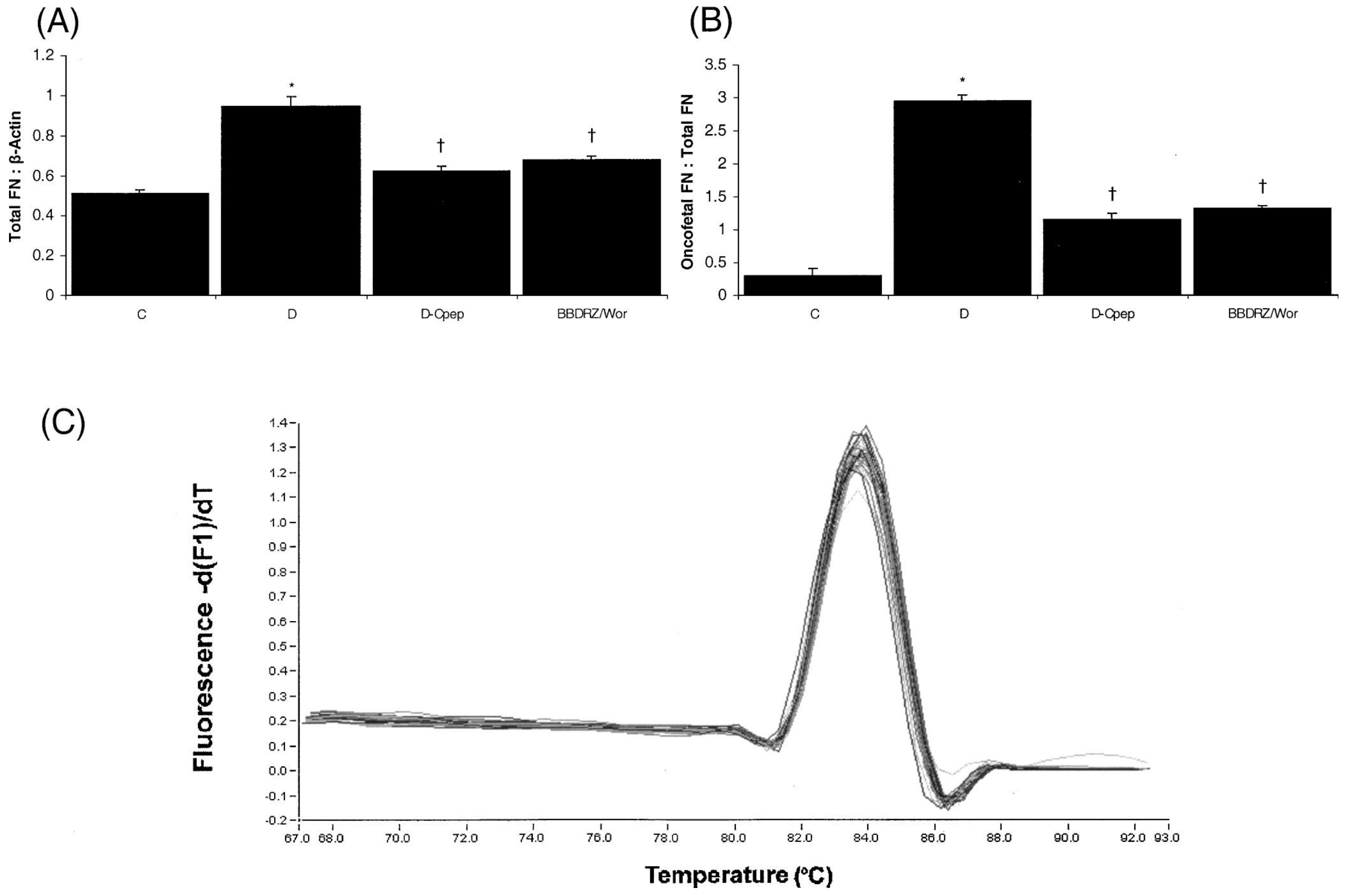

**FIGURE 1**

Effect of C-peptide on expression of FN isoforms in retina. Quantification of FN mRNA expression in retinal tissues by real time RT-PCR, showing (*A*) total FN and (*B*) oncofetal FN. Total FN mRNA levels are expressed as ratio of target mRNA to $\beta$-actin. Oncofetal FN levels are expressed as ratio of isoform mRNA to total FN mRNA. (*C*) Melting curve analysis (MCA) of oncofetal FN post-PCR products. MCA was used to determine specificity of PCR reactions (a single peak denotes specific dsDNA species in the reaction mixture). C = controls; D = BB/Wor rats; D-Cpep = BB/Wor diabetic rats treated with C-peptide; BBDRZ/Wor = BBDRZ/Wor-rats. $^{*}P < .05$ compared to controls; $^{\dagger}P < .05$ compared to BB/Wor animals; n = 6/group.

of both ET-1 and TGF-$\beta$ (Figure 2). C-peptide treatment of these diabetic animals failed to alter diabetes-induced ET-1 and TGF-$\beta$ expression (Figure 2). In addition, retinal tissues from BBDRZ/Wor diabetic rats exhibited increased levels of both ET-1 and TGF-$\beta$ transcripts as compared to control rats and increased ET-1 expression compared to type I BB/Wor diabetic rats. These findings suggest that C-peptide may regulate expression of oncofetal FN via ET-1 and TGF-$\beta$–independent mechanisms.

### C-PEPTIDE AND RETINAL CAPILLARY BM THICKENING

Increased expression of FN has been shown to be instrumental in mediating structural alterations and capillary BM thickening in retinas of diabetic rats (Evans et al., 2000). In both animal and human diabetes, we and others have demonstrated up-regulation of FN in target organs of chronic complications, including the retina (Evans et al., 2000; Roy et al., 1996). We examined whether C-peptide–mediated normalization of oncofetal FN was associated with prevention of capillary BM thickening in BB/Wor diabetic rats. Retinal tissues from animals were fixed in 2.5% gluteraldehyde and processed for electron microscopy. Retinal capillaries from the deep capillary bed were analyzed by orthogonal intercept method for BM thickening (Evans et al., 2000). C-peptide treatment was found to be ineffective in preventing diabetes-induced increases in retinal capillary BM thickening (Figure 3). Furthermore, type 2 BBDRZ/Wor diabetic animals exhibited BM thickening similar to that of untreated BB/Wor rats.

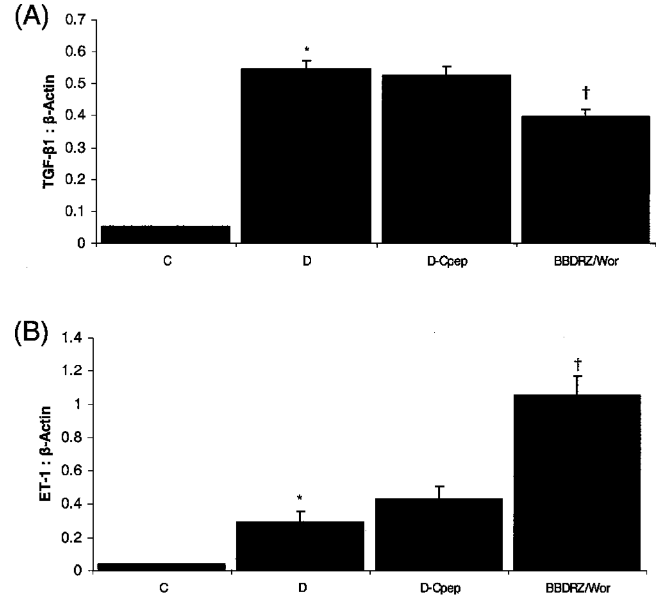

**FIGURE 2**
Effect of C-peptide on TGF-$\beta$1 and ET-1 expression. Quantification of fibrogenic growth factor expression in retinal tissues by real time RT-PCR quantification, showing (*A*) TGF-$\beta$1 and (*B*) ET-1 mRNA levels. TGF-$\beta$1 and ET-1 mRNA levels are expressed as ratio of target mRNA to $\beta$-actin. C = controls; D = BB/Wor rats; D-Cpep = BB/Wor diabetic rats treated with C-peptide; BBDRZ/Wor = BBDRZ/Wor-rats. $^{*}P < .05$ compared to control; $^{\dagger}P < .05$ compared to BB/Wor-animals; n = 6/group.

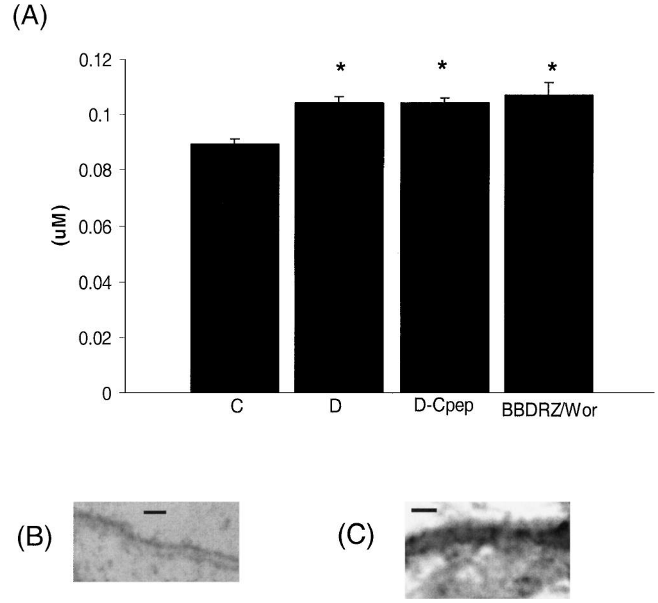

**FIGURE 3**
Effect of C-peptide on capillary basement membrane thickening. (*A*) Analysis of retinal basement membranes indicates a diabetes-induced increase in basement membrane thickening in both BB/Wor and BBDRZ/Wor rats after 8 months. C-peptide therapy was not effective in preventing diabetes-induced increased BM thickening. The lower panel shows representative electron micrographs of the retinal capillary from control (*B*) and diabetic rats (*C*). C = controls; D = BB/Wor rats; D-Cpep = BB/Wor diabetic rats treated with C-peptide; BBDRZ/Wor = BBDRZ/Wor-rats. $^{*}P < .05$ compared to control; n = 6/group. Bar = 230 nm.

### DISCUSSION

The mechanisms by which C-peptide mediates extracellular signals is not fully understood. Radioligand-binding studies in rats indicate that C-peptide binds to islet cells (Flatt et al., 1986). Furthermore, using more sophisticated fluorescence spectroscopy technique, human C-peptide was shown to bind to cell membrane preparations from various cell types, including renal tubular cells and vascular endothelial cells (Henriksson et al., 2001; Pramanik et al., 2001; Rigler et al., 1999). Recent studies also suggest that C-peptide possibly binds to cell surface receptors coupled to G-proteins (Johansson et al., 2002; Ohtomo et al., 1996; Shafqat et al., 2002). In support of such notion is the observation that C-peptide increases influx of extracellular calcium. This influx is completely abolished by treating cells with pertussis toxin. Furthermore, potential downstream mediators of C-peptide signaling seem to include members of MAPK pathway, extracellular signal-regulated kinase (ERK) 1 and 2 (Kitamura et al., 2003). Treatment of fibroblasts with C-peptide leads to phosphorylation of ERK 1/2, which is normalized by pertussis toxin and the MAPK inhibitor, PD098059. Furthermore, accumulating evidence suggests that C-peptide–mediated activation of ERK 1/2 and calcium influx could potentially cross-talk with PKC and PI3K activation (Kitamura et al., 2001). Furthermore, we have previously demonstrated that C-peptide increases autophosphorylation of the insulin receptor and increases the insulin receptor tyrosine kinase activity (Grunberger et al., 2001; Li et al., 2001, 2003). In addition, C-peptide increases insulin-receptor substrate-1 (IRS-1) phosphorylation and MAPK, and PI3K activities. These effects resulted in increased glycogen synthesis and amino acid uptake (Grunberger et al., 2001). These effects were diminished by wortmannin (PI3K and MAPK inhibitor), whereas pertussis toxin had no effects. These effects were induced by C-peptide alone and C-peptide potentiated the same effects by insulin, strongly suggesting that C-peptide has insulinomimetic effects (Grunberger et al., 2001; Li et al., 2003).

Many C-peptide effects, such as phosphorylation and increased activity of MAPK, PI3K, and PKC, are well characterized in the context of diabetic complications, including retinopathy. Such pathways could potentially arbitrate increased ECM protein deposition. However, our studies indicate that C-peptide reduces diabetes-induced oncofetal FN expression. Previous studies have shown that C-peptide activates eNOS and prevents diabetes-induced vascular permeability in the retina (Ido et al., 1997). It is plausible that C-peptide prevents vascular dysfunction. Our findings do support such a notion, in that C-peptide normalized oncofetal FN expression, which has been associated with activated endothelium but is ineffective against hyperglycemia-induced ET-1 and TGF-$\beta$ expression.

Our data demonstrate that C-peptide treatment was ineffective in reducing capillary BM thickening. In addition, BBDRZ/Wor rats showed higher BM thickness similar to that of untreated BB/Wor animals. It is of further interest to note that total and oncofetal FN levels were lower in the BB/Wor rats replenished with C-peptide as well as in BBDRZ/Wor rats compared to untreated BB/Wor rats. These data suggest that several other factors may be involved in capillary BM thickening, which may not be prevented by C-peptide therapy. This notion, however, needs to be confirmed with further experiments.

The effect of C-peptide on reduced oncofetal FN and total FN mRNA expression may have significant implications. FN is involved in the regulation of various cellular events through interaction with cell surface receptors (Kornblihtt et al., 1996). These cellular events include cell migration and proliferation of fibroblasts and endothelial cells. Expression of FN containing the EDB segment is highly regulated, being exclusive to embryonic and tumor tissues (Zardi et al., 1987). This oncofetal isoform of FN has recently been proposed to be an angiogenic marker. We have previously demonstrated that oncofetal FN is involved in proliferation of vascular endothelial cells (Khan et al., 2004). In addition, we have shown that oncofetal FN is up-regulated in vitreous specimens from patients with proliferative diabetic retinopathy. Our findings of reduced oncofetal FN expression in retinas of diabetic rats treated with C-peptide suggest potential novel avenues for the development of therapeutic modalities.

Our studies do indicate that C-peptide possesses important biological activity and is involved in regulating ECM protein composition. It is increasingly being realized that ECM is more than a mere bystander. The composition of the ECM is important in various physiological and pathological conditions and is a function of the tissue microenvironment (Badylak, 2002). In the context of proliferative diabetic retinopathy, the composition of ECM could potentially confer an angiogenic phenotype on vascular endothelial cells. Recent studies do support the notion that the composition and physical properties of ECM can modulate endothelial cell behavior, facilitating angiogenesis. Our studies indicate that C-peptide is able to reduce diabetes-induced oncofetal FN expression. We have previously demonstrated that oncofetal FN is involved in proliferation of endothelial cells (Khan et al., 2004). It is plausible that increased circulating C-peptide levels in patients with type 2 diabetes reduces oncofetal FN expression in the retina, which could offer some explanation of the disparity in the prevalence and progression to proliferative diabetic retinopathy in type 1 and type 2 diabetic patients (Keen et al., 2001).

### REFERENCES

Badylak, S. F. (2002) The extracellular matrix as a scaffold for tissue reconstruction. *Semin. Cell Dev. Biol.,* **13,** 377–383.

Brownlee, M. (2001) Biochemistry and molecular cell biology of diabetic complications. *Nature,* **414,** 813–820.

Cai, J., and Boulton, M. (2002) The pathogenesis of diabetic retinopathy: Old concepts and new questions. *Eye,* **16,** 242–260.

Cameron, N. E., Eaton, S. E., Cotter, M. A., and Tesfaye, S. (2001) Vascular factors and metabolic interactions in the pathogenesis of diabetic neuropathy. *Diabetologia,* **44,** 1973–1988.

Carnemolla, B., Balza, E., Siri, A., Zardi, L., Nicotra, M. R., Bigotti, A., and Natali, P. G. (1989) A tumor-associated fibronectin isoform generated by alternative splicing of messenger RNA precursors. *J. Cell Biol.,* **108,** 1139–1148.

Chen, S., Khan, Z. A., Cukiernik, M., and Chakrabarti, S. (2003) Differential activation of NF-kappa B and AP-1 in increased fibronectin synthesis in target organs of diabetic complications. *Am. J. Physiol. Endocrinol. Metab.,* **284,** E1089–E1097.

Evans, T., Deng, D. X., Chen, S., and Chakrabarti, S. (2000) Endothelin receptor blockade prevents augmented extracellular matrix component mRNA expression and capillary basement membrane thickening in the retina of diabetic and galactose-fed rats. *Diabetes,* **49,** 662–666.

Flatt, P. R., Swanston-Flatt, S. K., Hampton, S. M., Bailey, C. J., and Marks, V. (1986) Specific binding of the C-peptide of proinsulin to cultured B-cells from a transplantable rat islet cell tumor. *Biosci. Rep.,* **6,** 193–199.

Grunberger, G., Qiang, X., Li, Z., Mathews, S. T., Sbrissa, D., Shisheva, A., and Sima, A. A. F. (2001) Molecular basis for the insulinomimetic effects of C-peptide. *Diabetologia,* **44,** 1247–1257.

Henriksson, M., Pramanik, A., Shafqat, J., Zhong, Z., Tally, M., Ekberg, K., Wahren, J., Rigler, R., Johansson, J., and Jörnvall, H. (2001) Specific binding of proinsulin C-peptide to intact and to detergent-solubilized human skin fibroblasts. *Biochem. Biophys. Res. Commun.,* **280,** 423–427.

Ido, Y., Vindigni, A., Chang, K., Stramm, L., Chance, R., Heath, W. F., DiMarchi, R. D., Di Cera, E., and Williamson, J. R. (1997) Prevention of vascular and neural dysfunction in diabetic rats by C-peptide. *Science,* **277,** 563–566.

Jarnagin, W. R., Rockey, D. C., Koteliansky, V. E., Wang, S. S., and Bissell, D. M. (1994) Expression of variant fibronectins in wound healing: Cellular source and biological activity of the EIIIA segment in rat hepatic fibrogenesis. *J. Cell Biol.,* **127,** 2037–2048.

Johansson, J., Ekberg, K., Shafqat, J., Henriksson, M., Chibalin, A., Wahren, J., and Jörnvall, H. (2002) Molecular effects of proinsulin C-peptide. *Biochem. Biophys. Res. Commun.,* **295,** 1035–1040.

Johansson, B. L., Sjöberg, S., and Wahren, J. (1992) The influence of human C-peptide on renal function and glucose utilization in type 1 (insulin-dependent) diabetic patients. *Diabetologia,* **35,** 121–128.

Keen, H., Lee, E. T., Russell, D., Miki, E., Bennett, P. H., and Lu, M. (2001) The appearance of retinopathy and progression to proliferative retinopathy: The WHO Multinational Study of Vascular Disease in Diabetes. *Diabetologia,* **44 (Suppl),** S22–S30.

Khan, Z. A., Cukiernik, M., Gonder, J. R., and Chakrabarti, S. (2004) Oncofetal fibronectin in diabetic retinopathy. *Invest. Ophthalmol. Vis. Sci.,* **45,** 287–295.

Kitabchi, A. E. (1977) Proinsulin and C-peptide: A review. *Metabolism,* **26,** 547–587.

Kitamura, T., Kimura, K., Jung, B. D., Makondo, K., Okamoto, S., Canas, X., Sakane, N., Yoshida, T., and Saito, M. (2001) Proinsulin C-peptide rapidly stimulates mitogen-activated protein kinases in Swiss 3T3 fibroblasts: Requirement of protein kinase C, phosphoinositide 3-kinase and pertussis toxin-sensitive G-protein. *Biochem. J.,* **355,** 123–129.

Kitamura, T., Kimura, K., Makondo, K., Furuya, D. T., Suzuki, M., Yoshida, T., and Saito, M. (2003) Proinsulin C-peptide increases nitric oxide production by enhancing mitogen-activated protein-kinase-dependent transcription of endothelial nitric oxide synthase in aortic endothelial cells of Wistar rats. *Diabetologia,* **46,** 1698–1705, online, 30 Oct., 2003.

Kornblihtt, A. R., Pesce, C. G., Alonso, C. R., Cramer, P., Srebrow, A., Werbajh, S., and Muro, A. F. (1996) The fibronectin gene as a model for splicing and transcription studies. *FASEB J.,* **10,** 248–257.

Kornblihtt, A. R., Vibe-Pedersen, K., and Baralle, F. E. (1984) Human fibronectin: molecular cloning evidence for two mRNA species differing by an internal segment coding for a structural domain. *EMBO J.,* **3,** 221–226.

Li, Z. G., Qiang, X., Sima, A. A., and Grunberger, G. (2001) C-peptide attenuates protein tyrosine phosphatase activity and enhances glycogen synthesis in L6 myoblasts. *Biochem. Biophys. Res. Commun.,* **280,** 615–619.

Li, Z. G., Zhang, W., and Sima, A. A. (2003) C-peptide enhances insulin-mediated cell growth and protection against high glucose-induced apoptosis in SH-SY5Y cells. *Diabetes Metab Res. Rev.,* **19,** 375–385.

Nickeleit, V., Kaufman, A. H., Zagachin, L., Dutt, J. E., Foster, C. S., and Colvin, R. B. (1996) Healing corneas express embryonic fibronectin isoforms in the epithelium, subepithelial stroma, and endothelium. *Am. J. Pathol.,* **149,** 549–558.

Ohtomo, Y., Aperia, A., Sahlgren, B., Johansson, B. L., and Wahren, J. (1996) C-peptide stimulates rat renal tubular $Na^+$, K(+)-ATPase activity in synergism with neuropeptide Y. *Diabetologia,* **39,** 199–205.

Pierson, C. R., Zhang, W., and Sima, A. A. F. (2003) Proinsulin C-peptide replacement in type 1 diabetic BB/Wor-rats prevents deficits in nerve fiber regeneration. *J. Neuropathol. Exp. Neurol.,* **62,** 765–779.

Pramanik, A., Ekberg, K., Zhong, Z., Shafqat, J., Henriksson, M., Jansson, O., Tibell, A., Tally, M., Wahren, J., Jörnvall, H., Rigler, R., and Johansson, J. (2001) C-peptide binding to human cell membranes: importance of Glu27. *Biochem. Biophys. Res. Commun.,* **284,** 94–98.

Rigler, R., Pramanik, A., Jonasson, P., Kratz, G., Jansson, O. T., Nygren, P., Ståhl, S., Ekberg, K., Johansson, B., Uhlén, S., Uhlén, M., Jörnvall, H., and Wahren, J. (1999) Specific binding of proinsulin C-peptide to human cell membranes. *Proc. Natl. Acad. Sci. U. S. A.,* **96,** 13318–13323.

Roy, S., Cagliero, E., and Lorenzi, M. (1996) Fibronectin overexpression in retinal microvessels of patients with diabetes. *Invest. Ophthalmol. Vis. Sci.,* **37,** 258–266.

Samnegard, B., Jacobson, S. H., Jaremko, G., Johansson, B. L., and Sjöquist, M. (2001) Effects of C-peptide on glomerular and renal size and renal function in diabetic rats. *Kidney Int.,* **60,** 1258–1265.

Schwarzbauer, J. E., Tamkun, J. W., Lemischka, I. R., and Hynes, R. O. (1983) Three different fibronectin mRNAs arise by alternative splicing within the coding region. *Cell,* **35,** 421–431.

Shafqat, J., Juntti-Berggren, L., Zhong, Z., Ekberg, K., Kohler, M., Berggren, P. O., Johansson, J., Wahren, J., and Jörnvall, H. (2002) Proinsulin C-peptide and its analogues induce intracellular $Ca^{2+}$ increases in human renal tubular cells. *Cell Mol. Life Sci.,* **59,** 1185–1189.

Sheetz, M. J., and King, G. L. (2002) Molecular understanding of hyperglycemia's adverse effects for diabetic complications. *JAMA,* **288,** 2579–2588.

Sima, A. A. (2003) C-peptide and diabetic neuropathy. *Expert. Opin. Inves. Drugs,* **12,** 1471–1488.

Sima, A. A., Zhang, W., Sugimoto, K., Henry, D., Li, Z., Wahren, J., and Grunberger, G. (2001) C-peptide prevents and improves chronic type I diabetic polyneuropathy in the BB/Wor-rat. *Diabetologia,* **44,** 889–897.

Sjöquist, M., Huang, W., and Johansson, B. L. (1998) Effects of C-peptide on renal function at the early stage of experimental diabetes. *Kidney Int.,* **54,** 758–764.

The Diabetes Control and Complications Trial Research Group (1993) The effect of intensive treatment of diabetes on the development and progression of long-term complications in insulin-dependent diabetes mellitus. The Diabetes Control and Complications Trial Research Group. *N. Engl. J. Med.,* **329,** 977–986.

Tsilibary, E. C. (2003) Microvascular basement membranes in diabetes mellitus. *J. Pathol.,* **200,** 537–546.

UK Prospective Diabetes Study (UKPDS) Group (1998) Intensive blood-glucose control with sulphonylureas or insulin compared with conventional treatment and risk of complications in patients with type 2 diabetes (UKPDS 33). UK Prospective Diabetes Study (UKPDS) Group. *Lancet,* **352,** 837–853.

Wahren, J., Ekberg, K., Johansson, J., Henriksson, M., Pramanik, A., Johansson, B. L., Rigler, R., and Jörnvall, H. (2000) Role of C-peptide in human physiology. *Am. J. Physiol. Endocrinol. Metab.,* **278,** E759–E768.

Zardi, L., Carnemolla, B., Siri, A., Petersen, T. E., Paolella, G., Sebastio, G., and Baralle, F. E. (1987) Transformed human cells produce a new fibronectin isoform by preferential alternative splicing of a previously unobserved exon. *EMBO J.,* **6,** 2337–2342.

